# BanglaBlend: A large-scale nobel dataset of bangla sentences categorized by saint and common form of bangla language

**DOI:** 10.1016/j.dib.2024.111240

**Published:** 2024-12-20

**Authors:** Umme Ayman, Chayti Saha, Azmain Mahtab Rahat, Sharun Akter Khushbu

**Affiliations:** a Department of Computer Science and Engineering. Daffodil International University, Bangladesh; b Department of Information and Communication Technology, Comilla University, Bangladesh

**Keywords:** Bangla text classification, Bangla language, Text classification, Natural language processing

## Abstract

In the vibrant linguistic landscape of Bengali, spoken by millions in Bangladesh and India, the gap between saintly and common terms is culturally and computationally significant. Recognising this, we introduce BanglaBlend, a pioneering dataset created to capture these stylistic distinctions. BanglaBlend comes with 7350 annotated sentences, 3675 in saintly form and 3675 in common form, covering a crucial need in natural language processing (NLP) resources for Bangla. This dataset is transformational in a variety of applications. It contributes to the creation of NLP models that can detect and imitate Bengali stylistic nuances, hence improving tasks like as text categorisation, sentiment analysis, and style translation. BanglaBlend also facilitates literary analysis, cultural heritage projects, and the creation of domain-specific texts. To achieve the best data quality, rigorous pre-processing techniques such as anonymization and duplication removal were used. The style designations were extensively validated in three steps to ensure correctness. BanglaBlend is more than just a dataset; it is a cornerstone for future NLP research and development in Bangla. It is a valuable resource for studying stylistic diversity, aids in the development of context-aware language models, and is an essential tool for academic research and practical applications. By making BanglaBlend freely accessible, we hope to encourage cooperation and creativity within the Bangla NLP community, therefore adding to the worldwide variety of linguistic computational resources

Specifications Table

**Table:** 

Subject	Artificial Intelligence
Specific subject area	Machine Learning, Natural Language Processing, Bangla Text Classification
Type of data	Text Files (xlsx-formatted)
How data were acquired	The data for the BanglaBlend dataset was gathered from a variety of Bangla sources, including books, Facebook sites, literature, everyday conversations, educational content, and news stories from Bangladesh. This selected gathering technique was intended to confirm that data is distributed fairly between each saint and common form of Bangla language. The dataset contains 7350 sentences, with 3675 in the saint form and 3675 in the common form. Three steps of data validation were carried out to ensure high correctness and consistency, as well as the dataset's dependability for natural language processing applications. Native Bangla speakers thoroughly marked the data, and both writers checked it individually.
Data format	Text Files (xlsx-formatted)
Description of data collection	This selected gathering technique was intended to confirm that data is distributed fairly between each saint and common form of Bangla language. The dataset contains 7350 sentences, with 3675 in the saint form and 3675 in the common form. Three steps of data validation were carried out to ensure high correctness and consistency, as well as the dataset's dependability for natural language processing applications. Native Bangla speakers thoroughly marked the data, and both writers checked it individually.
Data source location	Publicly open Bangla books, Facebook pages, blogs, magazines and news articles
Data accessibility	Repository name: Mendeley Data Data identification number: 10.17632/7rx9mk8v4m.3 Direct URL to data: https://data.mendeley.com/datasets/7rx9mk8v4m/3

## 1 Value of the Data

• Distinguishing saint and common form of Bangla language is valuable to provide clarity and stylistic consistency that resolves the mixing issue of both forms in both writing and speaking, preserving cultural and historical context by recognizing the stylistic tones, and building several NLP applications such as text classification, automatic text translation, sentiment analysis, and so on, autoimatic content creation tools.Thus, the BanglaBlend dataset serves as a solid foundation for constructing and training NLP models that discriminate between saintly and common versions of Bengali sentences which is indispensable for many NLP applications.
• The dataset helps to create classifiers that can reliably assess the stylistic appearance of a given statement by categorizing it into Saint and Common forms. This improves the performance of text categorization systems, making them more accurate in tasks that require stylistic and contextual comprehension.
• BanglaBlend is a valuable resource for linguists and language scholars interested in investigating the stylistic and contextual features of Bangla language. It enables in-depth study and comparison of saint and common use patterns, which contributes to the larger subject of computational linguistics and stylistics.
• The dataset is an excellent educational resource for teaching and learning Bengali, particularly for understanding and practicing the proper usage of various stylistic formats. It may be included into language learning programs and educational platforms to give learners with realistic examples and exercises in both saintly and everyday settings.
• BanglaBlend enables the creation of equivalent datasets for other languages by establishing a standard for stylistic categorization in Bangla, enabling a more inclusive and thorough approach to NLP research across many linguistic settings.

## 2 Background

Millions of people speak Bangla, which has a rich linguistic legacy and cultural variety, yet there are no comprehensive resources that reflect the language's stylistic variants [1]. This gap impedes the development of sophisticated natural language processing (NLP) tools capable of comprehending and replicating the nuances of Bangla text. Recognising this requirement, BanglaBlend, a unique dataset, distinguishes between two key stylistic forms of Bengali: **saint (Sadhu Bangla)** and **common (Cholito Bangla)**. The more formal and archaic saint form in Bangla is usually found in religious texts, classical literature, and ceremonial settings. Because of its refined and conservative vocabulary and sentence structure, it is appropriate for formal or sacred works. On the other hand, Cholito Bangla, the common, modern form, is widely used in writing, media, and speech nowadays. It has evolved to fit the demands of everyday communication and is more straightforward and flexible. Sometimes native Bengali speakers blend these two stylistic forms and get confused to distinguish them, which creates ambiguity and misunderstanding, especially when interpreting Bengali texts in terms of speaking and writting. As a result, NLP tools can offer an automated stylistic discriminator or classifier to improve spoken and written communication, guaranteeing simplicity and clarity, maintaining cultural context, and fostering stylistic accuracy across many domains. Besides, stylistic diversity of Bengali, which ranges from religious literature to ordinary speech, is important in a variety of NLP applications such as literary analysis, sentiment recognition, automatic translator, text classification, and cultural preservation. Understanding these stylistic characteristics serves the theoretical motivation for developing NLP models that are not only accurate but also culturally and contextually sensitive. BanglaBlend was intended to provide a fair and representative collection of Bengali sentences. The dataset contains 7350 sentences, precisely classified into saint and common types. The categorisation procedure was thorough, with native Bengali speakers ensuring the annotations' correctness and consistency. This methodological rigour guarantees that BanglaBlend is a trustworthy resource for constructing and testing NLP models [2]. The dataset serves a variety of practical purposes, including text categorisation, literary analysis, cultural heritage, domain-specific text production, and creating AI based educational tools etc. BanglaBlend fills a significant void in the landscape of Bengali NLP resources. It establishes a standard for stylistic classification, encouraging the development of more comprehensive and complex NLP models. BanglaBlend's open availability aims to stimulate cooperation and creativity, supporting breakthroughs in the interpretation and processing of the Bangla language and its stylistic richness.

### 2.1 Data description

Bangla, considered to be among the most noteworthy Eastern Indo-Aryan languages on the Indian subcontinent, ranks as the seventh most spoken language worldwide, with around 272.8 million native speakers [3]. As Bangladesh's official language and one among India's 22 designated languages, Bengali retains enormous cultural and linguistic importance, with a substantial global speaker community [4]. This language presents substantial issues for natural language processing (NLP) applications such as text categorisation, literary analysis, cultural heritage, and domain-specific text production, as there are no comprehensive resources that reflect the language's stylistic variants. To mitigate this gap, we have developed the 'BanglaBlend' dataset, an exquisitely curated collection of 7350 sentences gathered fairly to do this study. The dataset is available as “BanglaBlendCleanedData.xlsx”, the cleaned data file, in the repository.

The BanglaBlend dataset was compiled from a variety of publicly available Bangla novels, Facebook pages, magazines, blogs, and news items, assuring a varied representation of modern language use. Maintaining an equal proportion of expressions over two categories, Saint and Common, was crucial to the dataset's curation. This balanced representation is vital for developing models that can effectively generalize across language structures and complexity.

**Fig. 1:**
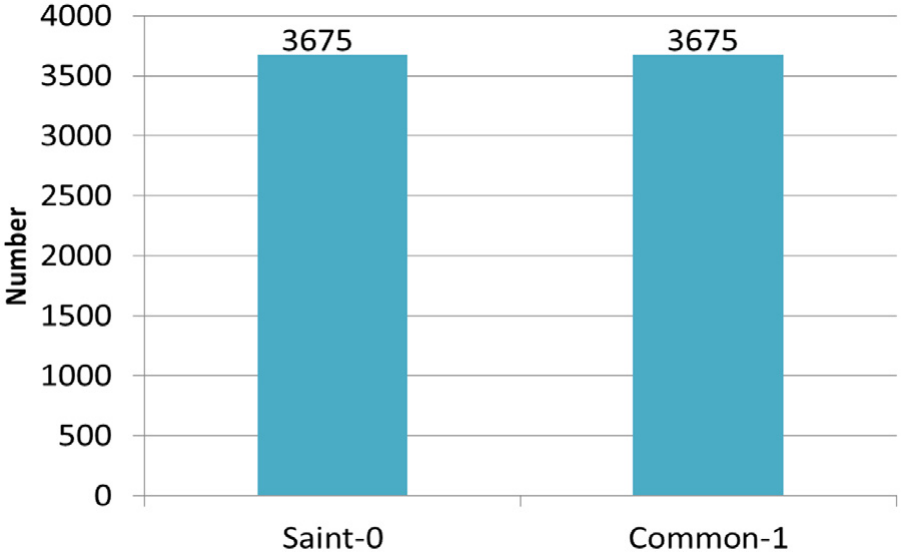
Class distribution of saint and common form of language.

The following description gives a complete summary of the variables in the BanglaBlend dataset, allowing researchers to fully leverage this resource for developing NLP applications in Bangla. Table 1 describes the dataset structure, which includes the various components and their annotations, making it easier to utilize for study and development. Fig. 1 depicts the class distribution across the two Bangla language groups in the dataset: 3675 sentences (50 %) in saint form and 3675 phrases (50 %) in common form.

**Table 1:** Dataset description with attributes and possible values.

**Fig. 2:**
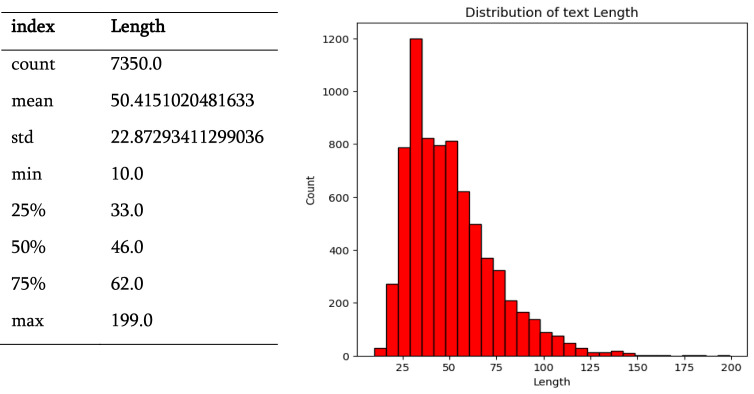
The frequency distribution of Bangla Text length for the dataset.

**Fig. 3:**
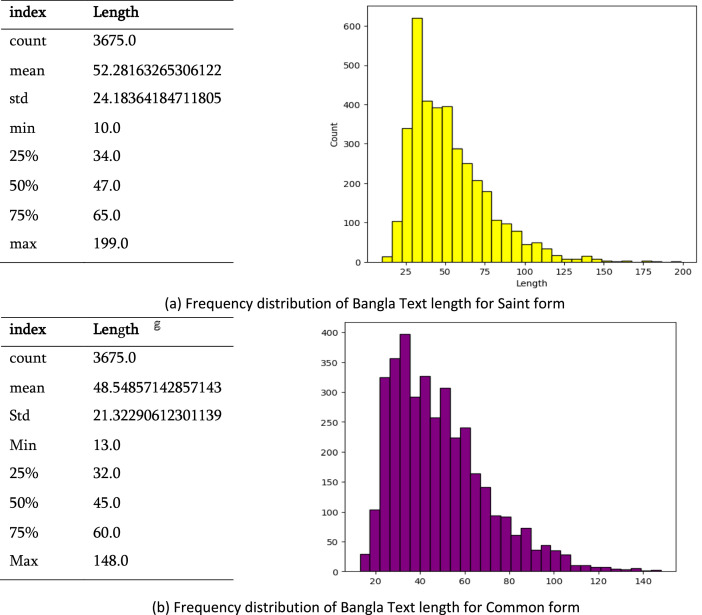
The frequency distribution of Bangla Text length for Saint and Common forms (a, b).

Fig. 2 depicts the text length distribution within the BanglaBlend dataset, Fig. 3 depicts the text length distribution separately for into common and frequent forms. There are 3675 saint sentences, with an average length of 52.28 characters and a standard deviation of 24.18 characters. These sentences range in length from 10 to 199 characters, with the middle 50 % lying between 34 and 47 characters and a median of 65 characters. The common category comprises 3675 sentences, with an average length of 48.55 characters and a standard deviation of 21.32 characters. Sentence lengths range from 13 to 148 characters, with the middle 50 % falling between 32 and 45 characters and having a median length of 60 characters.

Overall, the BanglaBlend dataset reveals a balanced proportion of sentences in both formats. The diversity in sentence lengths is comparable across categories, with the saint category having the largest maximum sentence length. This thorough statistical summary aids in understanding the dataset's features, which are critical for constructing and verifying models for NLP applications in Bengali.

**Fig. 4:**
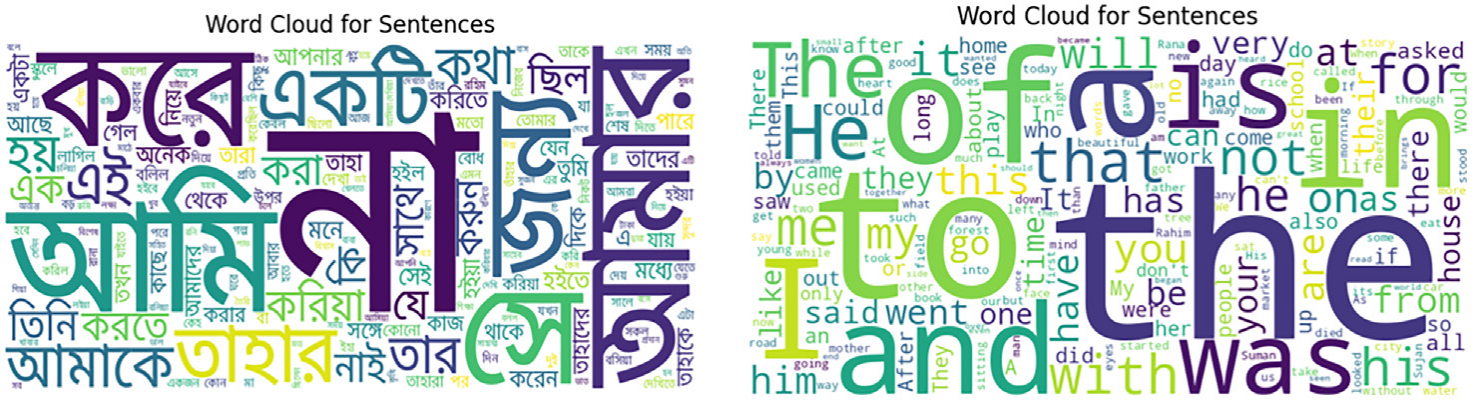
Word cloud for all words (in Bengali and in English) within BanglaBlend dataset.

Table 2 presents the 25 most common words from the BanglaBlend dataset, along with their frequencies. “” appears most frequently (771 times), highlighting its common use in all sentences. Besides Fig. 4 represents word clouds in Bangla and English terms from the BanglaBlend dataset, which graphically illustrates the frequency and prominence of terms inside their appropriate sentence form based on their appearance in the dataset.

**Table 2:** Most common 25 words with their frequency.

However, the study contains stop words, which are popular words with little significance and are normally omitted from text analysis. Their inclusion may not provide a complete view of the datasetʼ most important keywords [5]. Despite this constraint, studying high-frequency terms gives useful information about the dataset's common lexicon and linguistic trends. This understanding is critical for designing successful language models and algorithms, which improve prediction and classification accuracy. Furthermore, focusing on these terms aids in crucial data preparation activities like, removal of stop-words and selection of features, which help to refine this dataset for training Natural Language Processing (NLP) models.

### 2.2 Experimental design, materials and methods

To create a large number of text data, particularly in Bangla, which presents considerable obstacles, the BanglaBlend dataset production procedure takes an organized approach. Initially, Bangla material was gathered from publicly accessible sources such as books, blogs, Facebook pages, magazines, and news items. The material was organized into a Google Spreadsheet file for aggregation. Subsequently, the dataset underwent stringent preparation stages, including initial cleaning, anonymization, removal of duplication, and also filtering out any occurrences of profanity.

**Fig. 5:**
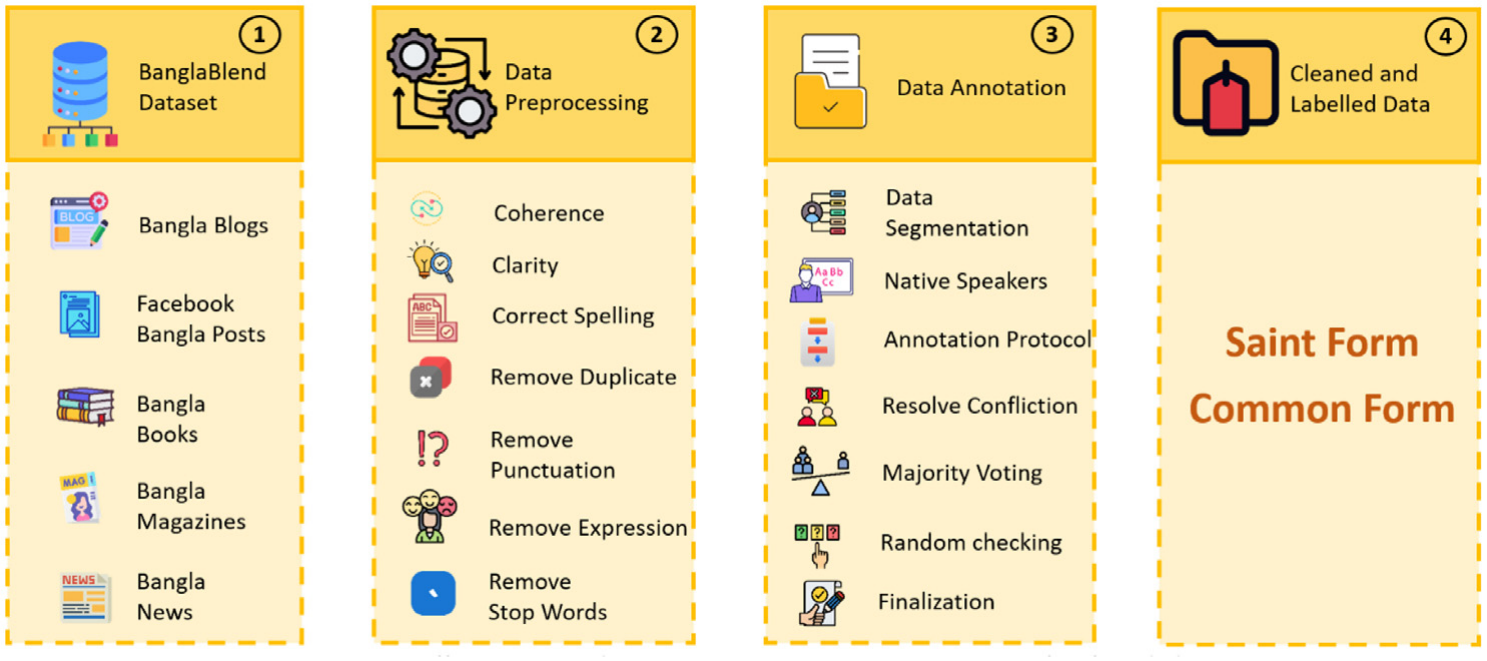
Data collection and preprocessing to create BanglaBlend dataset.

In this following stage, native Bangla speakers meticulously evaluated the dataset in three phases. In each phase, assessors individually labelled texts using two unique forms: saint and common. This careful evaluation confirmed that sentences were correctly classified, increasing the dataset's dependability and quality. Fig. 5 depicts the methodical workflow of the dataset production process, including each stage from data collection to final labelling by native speakers.

After acquiring the raw Bangla text, the BanglaBlend dataset is processed via many important phases to assure its quality and usefulness. Initially, primary cleaning is done to improve coherence, clarity, and word spelling. Any errors discovered during this step are manually corrected or deleted in order to ensure data integrity. Next, duplicate values are found and eliminated to prevent repetition and guarantee that each entry is unique. Punctuation marks, which are frequently irrelevant to linguistic research, are deleted to simplify the text data. Regular expressions are used to find and delete undesired patterns in the text, hence improving the dataset. Finally stop words are removed. These extensive pretreatment methods jointly guarantee that the BanglaBlend dataset is clean, accurate, and ready for further analysis and modelling. Algorithm 1 depicts the data annotation process using Inter Annotator Agreement (IAA) [6]. Finally, we acquire cleaned and labelled data categorised as Saint and Common form.

Algorithm 1 BanglaBlend data annotation process.

**Table:** 

Input:	Raw Bangla Sentence, B_Sentence_
Output:	Form of Bangla Sentence, B_Form_
Step-1:	Take all of the sentences for annotation
Step-2:	Select a group of native Bengali speakers (e.g., 3 annotators in 3 step) having strong language proficiency
Step-3:	Assign each sentences to multiple annotators (e.g., 3 annotators per sentence) to ensure redundancy
Step-4:	Use ‘Google Spreadsheet’ where annotators can label each sentence as saint, or common
Step-5:	Calculate agreement using statistical measures (i.e., Fleiss' Kappa) to calculate the inter-annotator agreement (IAA)
Step-6:	Calculate Fleiss’ Kappa:
Step-7:	$FK=\frac{\bar{A}-{\bar{A}}_{e}}{1-{\bar{A}}_{e}}\;\left\{ \begin{array}{c} Where,\;\bar{A}\;is\;the\;mean\;of\;the\;observed\;agreement,\;\\ {\bar{A}}_{e}\;is\;the\;mean\;of\;the\;expected\;agreement\;by\;chance \end{array}\right.$
Step-8:	Check conflict of resolution, sort them for further review and annotate with expert annotator
Step-9:	Measure majority voting for each form and set the final annotation with the highest vote
Step-10:	Calculate the confidence score for each sentence based on the proportion of annotators who agreed on the label
Step-11:	$Confidence\;Score=\frac{Number\;of\;AnnotatorsAgreeing}{Total\;Number\;of\;Annotators}\;\{\;A\;high\;confidence\;score\;indicates\;the\;high\;reliablity$
Step-12:	Check randomly samples annotated sentences and perform a quality check to ensure annotations meet the required standards
Step-13:	Compile the final annotated sentences into the BanglaBlend dataset

The accuracy of five deep learning models in categorising text data into two categories, 'Saint' and 'Common', was find out for this dataset. 50 epochs were used to train all of the models separately, where epochs represent a complete run over the entire dataset. The maximum number of batches was set to 64, which meant that after examining 64 samples, the model would adjust its weights. A comparison study was performed to assess the performance of LSTM, bi-LSTM, Conv1D, combined Conv1D-LSTM-based model, and combined Conv1D-Bi-LSTM-based model, shown in Table 3. Bi-LSTM Based Model attained the best accuracy (92.65 %).

**Table 3:** Performance of deep learning models on our BanglaBlend dataset.

Model	Class	Precision(%)	Recall(%)	F1-Score(%)	Accuracy(%)
LSTM	Saint	93.12	88.56	90.78	91.02
	Common	89.11	93.47	91.23	
Bi-LSTM	Saint	93.35	91.83	92.58	92.65
	Common	91.98	93.48	92.72	
Conv1D	Saint	88.33	90.49	89.40	88.84
	Common	90.14	87.19	88.64	
Conv1D-LSTM	Saint	89.22	87.71	88.45	88.57
	Common	87.94	89.37	88.64	
Conv1D-Bi-LSTM	Saint	88.40	86.96	87.67	87.76
	Common	87.13	88.56	87.84	

### 2.3 Data collection

In order to guarantee through representation, the BanglaBlend dataset was assembled by the Bangla text collected from various sources. Specifically, the texts were collected from books, Facebook posts, literature works, daily conversation, news articles, blogs and educational contents in Bengali. A well-rounded linguistic representation was intended by this methodical selection approach, which balanced the dataset equally between the “saint” and “common” versions of the Bangla language. The dataset has 7350 sentences total, divided equally between 3675 sentences in each type. The sentences in “saint” category were accumulated from Bengali literature works written in saint form specifically to ensure the authenticity. Besides, the sentences in “common” category were collected from social media posts and comments, daily conversation, news articles, blogs.

### 2.4 Data annotation

To annotate a dataset of Bengali sentences, a systematic approach is employed. Initially, every sentence is allocated to a group of proficient native Bengali speakers (three annotators per sentence). Using Google Spreadsheet, numerous annotators mark each sentence to ensure repetition and sentences are classified as “saint” or “common” by following the rules of Bengali grammar. By comparing the observed agreement to the expected agreement, statistical methods such as Fleiss' Kappa are used to measure the inter-annotator agreement (IAA). When labels dispute, the sentences are examined and reconciled by a professional annotator. The final annotation is determined by majority voting; higher scores indicate greater dependability. The confidence score is computed based on the percentage of annotators that agree on a given label. Samples are subjected to random quality tests to make sure the annotations adhere to the necessary requirements. After completion, the annotations are combined and stored in the “BanglaBlend.xlsx” dataset for later use.

### 2.5 Limitations

The BanglaBlend dataset, while useful for Bengali NLP, has a few drawbacks. The crucial fact can be said that this dataset may not be used for researches on other languages as the English translation for both saint and common sentences are same. Manual labelling by native speakers in three phases causes bias and inconsistency. The dataset's sources (books, blogs, Facebook pages, magazines, and news items) may not completely represent the diversity of Bangla language usage, limiting generalisability. Pre-processing procedures may mistakenly eliminate valuable information with noise. Additionally, focussing just on saint and common may oversimplify the complexity of Bengali sentences, ignoring sophisticated grammatical patterns. Despite these shortcomings, the BanglaBlend dataset is an important resource for Bengali NLP research.

### 2.6 Ethics statement

The BanglaBlend dataset prioritises ethical data collecting methods. Publicly available information from books, blogs, Facebook pages, magazines, and news items was obtained and used appropriately. Strict criteria guaranteed that only copyright-free material was included, reducing the possibility of infringement. Furthermore, the dataset adheres to responsible usage guidelines, ensuring no harm or infringement of person rights. Adherence to Facebook's content usage regulations ensured that no further permissions were needed for content collected from their pages.

## CRediT authorship contribution statement

**Umme Ayman:** Conceptualization, Data curation, Visualization, Software, Resources, Writing – review & editing. **Chayti Saha:** Data curation, Methodology, Writing – original draft, Visualization, Investigation. **Azmain Mahtab Rahat:** Data curation, Validation. **Sharun Akter Khushbu:** Supervision.

## Data Availability

Mendeley DataBanglaBlend: A Large-Scale Nobel Dataset of Bangla Sentences Categorized by Saint(Sadhu) and Common(Cholito) Form of Bengali Language (Original data).
